# Theoretical Evaluation of Multi-Breed Genomic Prediction in Chinese Indigenous Cattle

**DOI:** 10.3390/ani9100789

**Published:** 2019-10-11

**Authors:** Lei Xu, Zezhao Wang, Bo Zhu, Ying Liu, Hongwei Li, Farhad Bordbar, Yan Chen, Lupei Zhang, Xue Gao, Huijiang Gao, Shengli Zhang, Lingyang Xu, Junya Li

**Affiliations:** 1 Institute of Animal Sciences, Chinese Academy of Agricultural Sciences, Beijing 100193, China; xuleirock@163.com (L.X.); wangzezhao1@163.com (Z.W.); zhubo@caas.cn (B.Z.); yliu2333@sina.com (Y.L.); lihongweicaas@163.com (H.L.); farhadnevergiveup@yahoo.com (F.B.); chenyan0204@163.com (Y.C.); zhanglupei@caas.cn (L.Z.); gaoxue76@126.com (X.G.); gaohj111@sina.com (H.G.); 2 Institute of Animal Husbandry and Veterinary Research, Anhui Academy of Agricultural Sciences, Hefei 230031, China; 3 National Engineering Laboratory for Animal Breeding, Key Laboratory of Animal Genetics, Breeding and Reproduction, Ministry of Agriculture, College of Animal Science and Technology, Chinese agriculture University, Beijing 100193, China; zhangslcau@cau.edu.cn

**Keywords:** genomic prediction, linkage disequilibrium, resampling approaches, Chinese indigenous cattle

## Abstract

**Simple Summary:**

In order to evaluate the potential application of genomic selection (GS) for Chinese indigenous cattle, we assessed the influence of combining multiple populations on the reliability of genomic predictions for 10 indigenous breeds of Chinese cattle using simulated data. We found the predictive accuracies to be low when the reference and validation populations were sampled from different breeds. When using multiple breeds for the reference population, the predictive accuracies were higher if the reference was comprised of breeds with close relationships. In addition, the accuracy increased in all scenarios when the heritability increased, and the genetic architecture of the QTL can affect genomic prediction. Our study suggested that the application of meta-populations can increase accuracy in scenarios with a reduced size of reference populations.

**Abstract:**

Genomic selection (GS) has been widely considered as a valuable strategy for enhancing the rate of genetic gain in farm animals. However, the construction of a large reference population is a big challenge for small populations like indigenous cattle. In order to evaluate the potential application of GS for Chinese indigenous cattle, we assessed the influence of combining multiple populations on the reliability of genomic predictions for 10 indigenous breeds of Chinese cattle using simulated data. Also, we examined the effect of different genetic architecture on prediction accuracy. In this study, we simulated a set of genotype data by a resampling approach which can reflect the realistic linkage disequilibrium pattern for multiple populations. We found within-breed evaluations yielded the highest accuracies ranged from 0.64 to 0.68 for four different simulated genetic architectures. For scenarios using multiple breeds as reference, the predictive accuracies were higher when the reference was comprised of breeds with a close relationship, while the accuracies were low when prediction were carried out among breeds. In addition, the accuracy increased in all scenarios with the heritability increased. Our results suggested that using meta-population as reference can increase accuracy of genomic predictions for small populations. Moreover, multi-breed genomic selection was feasible for Chinese indigenous populations with genetic relationships.

## 1. Introduction

Genomic prediction has become a widely used strategy for selection of candidate animals based on the estimation of genomic estimated breeding values (GEBV) [1]. Genomic selection can promote genetic progress, increase selection accuracy, and reduce the generation interval [1,2]. Many previous studies have suggested the estimated accuracy of GEBV for training populations without phenotypes mainly depends on the population size and the extent of linkage disequilibrium (LD) between markers and quantitative trait loci (QTL s) [3,4,5]. In reality, a large reference population is difficult to construct, especially for indigenous breeds with limited population size [6,7,8]. Thomasen et al. suggested a negative impact of small size reference population on the reliability of genomic prediction [9].

To avoid low estimated power from limited size of the training population, one possible resolution is to generate a large reference population by pooling multiple breeds into one meta-population. However, this strategy was based on the assumption that the effects of single nucleotide polymorphisms (SNPs) were constant across breeds [10]. Several studies have evaluated the adequacy of different pooling strategies for the training and validation sets in multiple breeds using both simulation and real data. For instance, one recent simulated study suggested that admixed data can be used to effectively predict purebred performance when target breeds were included in the training data set [11]. Hozé et al. showed an improvement of 2.9% in prediction accuracy in multi-breed populations (Holstein-Normande-Montbéliarde) compared to single breed prediction [12]. Moreover, Jónás et al. observed a maximum gain of 8% and 5% in the Abondance and Simmental breeds using a mixed reference population [13]. In contrast, Kachman et al. observed that multi-breed as training population did not increase prediction accuracies compared to single breed analysis when enough animals are available in each breed. In general, predictive accuracy is relatively high when the relationship of subpopulations are genetically close [14,15]. Therefore, prediction using multiple population with small size may be affected by the genetic relationships, consistency of LD phase and common QTLs among breeds.

Simulating realistic genotypes and phenotype can be used to evaluate the prediction of breeding strategies. Most current simulation methods for selection and evolutionary processes can be divided into forward in time [16,17,18,19] and backward in time through coalescent theory [20,21,22]. However, these approaches cannot be directly utilized to explore the multiple breed with a population-specific LD pattern. Therefore, Chen et al. proposed a resampling approach to simulate a genome-wide genotype with a population-specific LD pattern [23,24], this approach can simulate a genotype from the real population, and reflect the allele frequencies and population LD pattern manifested in real population, which are most appropriate to investigate the genomic prediction in multiple populations.

Chinese indigenous cattle have a diverse LD pattern, thus investigation of these cattle can offer valuable insights into elucidating the genetic basis underpinning important traits and evaluating the efficiency of the potential application of multi-breed genomic selection [25]. Many indigenous cattle have relatively small population size, thus implementation of genomic selection for multiple populations is necessary and technically feasible in Chinese indigenous cattle. The objective of this study is to evaluate the efficiency of the potential application of multi-population genomic selection in Chinese indigenous cattle, and investigate a feasible genomic selection strategy for Chinese indigenous cattle with a small population size. We also evaluated the effect of heritability and genetic architecture on a multi-breed genomic prediction.

## 2. Material and Methods

### 2.1. Animals and Genotype Quality Control

The genotype data were retrieved from our previous study [26]. All individuals from 10 Chinese cattle breeds (Supplementary Table S1) were genotyped by the Illumina BovineHD Beadchip (Illumina, Inc., San Diego, CA). Inner Mongolia cattle (MGC, n = 21), Yanhuang cattle (YHC, n = 24), Caidamu cattle (CDM, n = 25), Xizang cattle (XZC, n = 26), Pingwu cattle (PWC, n = 24), Liangshan cattle (LSC, n = 22), Zhaotong cattle (ZTC, n = 23), Wenshan cattle (WSC, n = 24), Hannan cattle (HNC, n = 26), and Nandan cattle (NDC, n = 25).

SNP quality control (QC) was conducted using PLINK v1.9 [27]. Samples with total call rates < 0.90 were removed, and only SNPs located on autosomes were considered for subsequent analyses. SNPs with call rates (CR) < 0.90, minor allele frequencies (MAF) <0.01 and that deviated significantly from Hardy-Weinberg Equilibrium (*p* < 1.0 × 10^−6^) were excluded. After QC, the genotype was phased with BEAGLE v5.0 [28], and 10 Chinese indigenous cattle populations were divided into diverse groups by K-means cluster implemented in R program [29,30].

### 2.2. Simulation of Genotypes

The simulation procedure was set up to generate the similar linkage disequilibrium structure of studied breed as described by previous study [26]. We started with 21–26 available samples for each breed comprising of 658,234 SNPs. For each breed, we simulated 1500 individuals by resampling approach, which assumes a block of 500 adjacent markers for each population. Thus, the simulated data can retain the similar LD patterns (broken by strong recombination hotspots) and allele frequencies as observed in the real data.

### 2.3. Principal Component Analysis and Persistence of Allele Phase

To investigate the genomic composition of the real and simulated populations, the principal components and the genomic relationship matrix (GRM) [31] were calculated using high quality SNPs. Principal components were estimated using the *prcomp* function implemented in R package “*stats*”.

We assessed the persistence of allele between the real genotypes and simulated genotypes. The persistence of phase was measured by the Pearson correlation between the average means of linkage phase in different distances. The correlation coefficients (*r*) were computed across pair-wise markers between populations, a series of marker distance intervals were set to bins of 2.5 kb for small distance (0–10 kb), 10 kb for medium distance (10–100 kb) and 100 kb for a large distance (100–1000 kb).

### 2.4. Simulation

Phenotypes were simulated based on simulated genotype. A range of scenarios were simulated as described in Table 1, which include various heritabilities, numbers of QTL, and distribution of QTL effects. A set of SNP markers were randomly selected as QTLs. Subsequently, their additive effects were sampled from three types of normal distribution: N (0,0.0001 $\sigma_{g}^{2}$), N (0,0.001 $\sigma_{g}^{2}$), N (0,0.01 $\sigma_{g}^{2}$), which present large, medium, and small effect QTLs, and $\sigma_{g}^{2}$ is the additive genetic variance. 

The true genetic values were calculated as the sum of the effects of their genotype for the QTL. Environmental effects were randomly drawn from a normal distribution with a mean of 0 and variance = Vg(1−h2)h2 where *v*_g_ is the variance of the genetic values and h^2^ is heritability of trait. Phenotypes for the individuals were obtained by summing the genetic and environmental effects.

Phenotypes were simulated for each scenario, a residual drawn from a Gaussian distribution with appropriate variance to generate three traits with heritability 0.1, 0.3, and 0.6, respectively. All scenarios were replicated 10 times.

True breeding values (TBVs) were calculated as the sum of the effects of their genotype for the QTLs as the formula

(1) $$\mathrm{TBV}=\overset{n}{\underset{j=1}{\sum }}x_{ij}a_{j}$$

where $x_{ij}$ is the genotype of individual *j* coded as 0, 1, and 2 for QTL *i*; $a_{j}$ is the additive effect of QTL *i*; and *n* is the number of QTL.

### 2.5. Genomic Evaluation

Breeding values were estimated for all scenarios using genomic best linear unbiased prediction (GBLUP). The following model was fitted for GBLUP.

(2) $$y=X_{b}+Z_{a}+G_{g}+e$$

where **y** is a vector of phenotypes, ***X***, ***Z***, and ***G*** are design matrices allocating phenotypes to vectors ***b***, ***a***, and ***g***, with fixed effects (overall mean and breed), polygenic breeding values based on genomic breeding values, respectively, and ***e*** is a vector of residual errors distributed as N(0, I $\sigma_{e}^{2}$), with identity matrix I and error variance $\sigma_{e}^{2}$. Polygenic and genomic breeding values were distributed as N(0, A $\sigma_{a}^{2}$) and N(0, GRM $\sigma_{g}^{2}$), respectively, where A is a numerator relationship matrix, $\sigma_{a}^{2}$ is the additive genetic variance, GRM is a genomic relationship matrix, and $\sigma_{g}^{2}$ is the genetic variance explained by genomic variants. The GRM was constructed following Yang et al. [32].

### 2.6. Reference and Validation Populations

Three scenarios of references were considered based on the size and composition of reference population.

Scenario I: Single breed, the reference population comprised 1200 individuals of one simulated breed. Each breed was separately used as reference population.

Scenario II: The reference population comprised 1200 randomly selected individuals from three simulated breeds, and with the same number of animals selected from each population. Because 10 populations were divided into three groups according to K-means cluster and Principal Component Analysis (PCA)27, we combined the three groups into three types of reference populations. For comparison, the fourth reference population was comprised of three breeds (XZC, LSC, HNC) from different groups.

Scenario III: Combined 10 breeds, one reference population was comprised of 1200 individuals with 120 randomly selected individuals from each of 10 populations.

### 2.7. Accuracy of Genomic Prediction

The accuracies of genomic prediction were estimated from the correlation between the predicted genetic value and TBV of the simulated phenotypes. Each case of simulation was replicated five times and the mean accuracy was calculated.

## 3. Results

### 3.1. Simulation of Genotype

**Figure 1:**
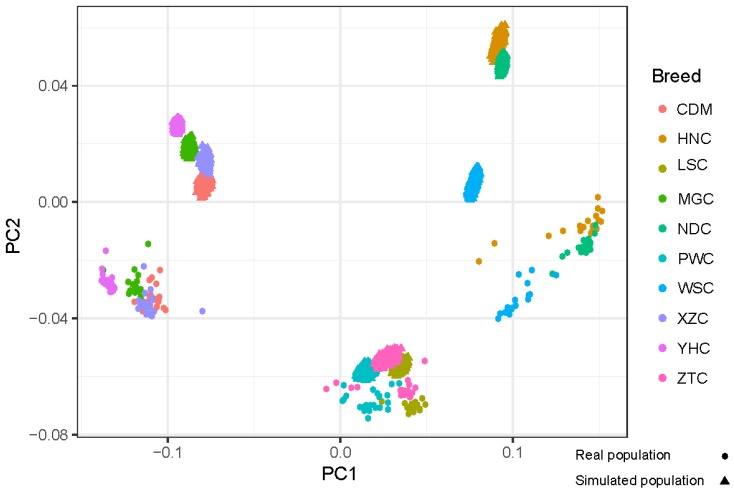
Principal component analysis of the real and simulated populations. MGC = Inner Mongolia cattle, YHC = Yanhuang cattle, CDM = Caidamu cattle, XZC = Xizang cattle, PWC = Pingwu cattle, LSC = Liangshan cattle, ZTC = Zhaotong cattle, WSC = Wenshan cattle, HNC = Hannan cattle, and NDC = Nandan cattle.

**Figure 2:**
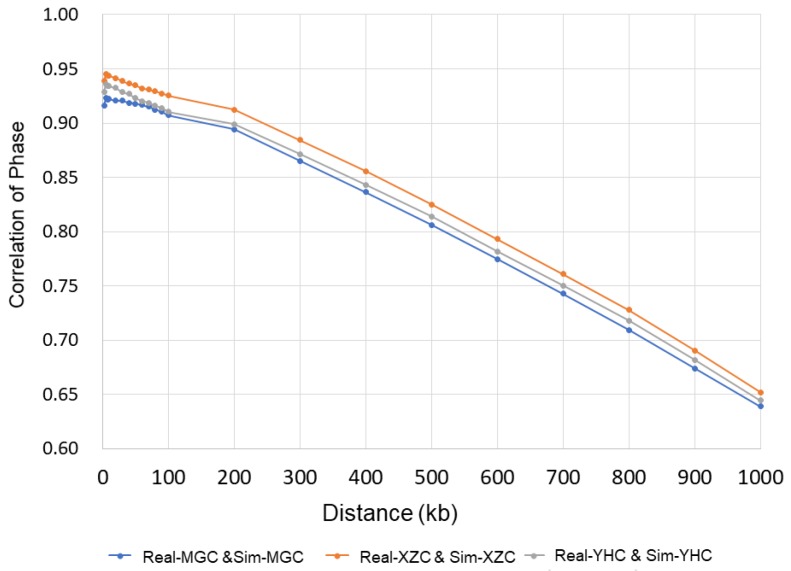
The persistence of allele phase between real and simulated genotype for breed Inner Mongolia Cattle (MGC), Xizang cattle (XZC), and Yanhuang cattle (YHC).

To evaluate the performance of the simulation analysis, we investigated the genomic composition and genetic structure of the real and simulated populations by PCA and persistence of allele phase. The PCA result of simulated analysis was generally consistent with real data (Figure 1), and the simulated population can be divided into three groups using K-means cluster, which included group NCC (MGC, YHC, CDM, and XZC), group SWC (PWC, LSC, and ZTC), group SCHC (HNC, NDC, and WSC). The overall correlation of phase between markers in real and simulated genotypes for each population were high as expected. Phase correlations between SNPs decreased from 0.94, 0.93 and 0.92 (distances of 0–2.5 kb) to 0.83, 0.81, and 0.81 (distances of 400–500 kb) of the real and simulated genotypes for XZC, YHC, and MGC, respectively (Figure 2).

### 3.2. Prediction with Different Reference Population

#### 3.2.1. Prediction with Single Breed Reference

**Figure 3:**
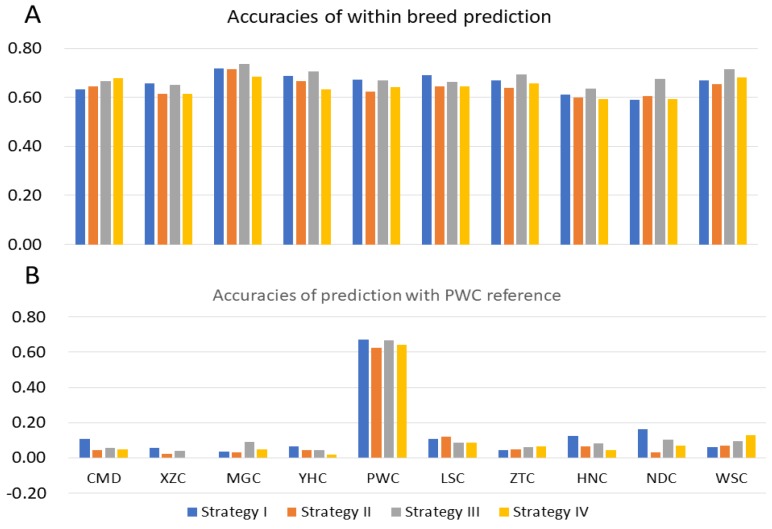
Accuracies of genomic prediction within breeds and among breeds for four traits with high heritability (0.6). (**A**) Accuracies of within-breed prediction; (**B**) Accuracies of prediction with Pingwu cattle (PWC) reference.

In scenario I with heritability of 0.6, single breed reference population was used to predict the GEBVs for 10 breeds. Figure 3 showed the accuracies of within-breed (A) and among-breed (B) prediction for 10 breeds using PWC as reference. To estimate the influence of genetic architecture on accuracy of genomic prediction, we performed simulation analysis considering four different strategies. The average predictive accuracies were larger within breeds compared to across breeds. For instance, we found the average predictive accuracies for PWC were 0.67, 0.62, 0.67, and 0.64 for four traits, while 0.08, 0.05, 0.07, and 0.06 were observed for other breeds. Similar results were found when considered each of the other nine breeds as the reference population. The average predictive accuracies for 10 breeds were 0.66, 0.64, 0.68, and 0.64 for four simulation strategies for within-breed estimation, the accuracies across breeds were about 0.07 (Supplementary S2).

#### 3.2.2. Admixed Breeds Reference

**Figure 4:**
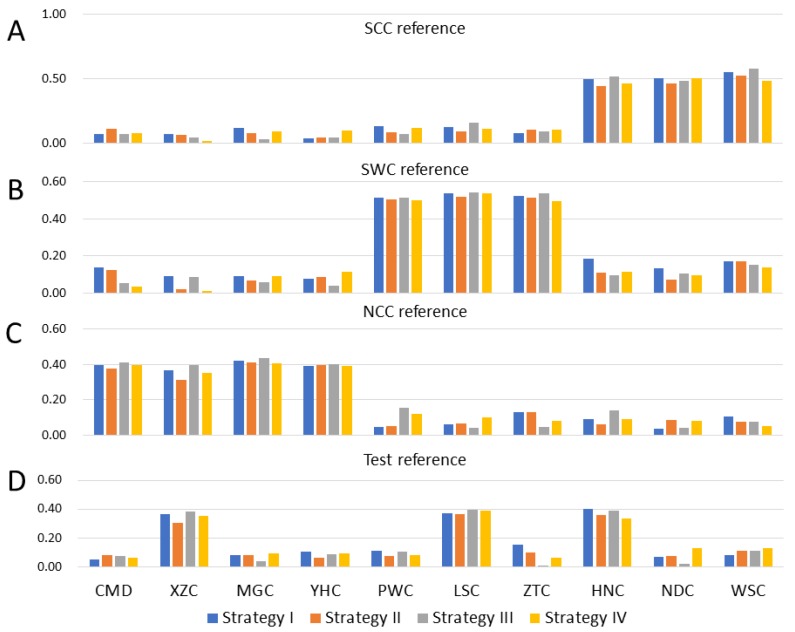
Accuracies of genomic prediction for four traits with heritability (0.6) in 10 breeds. (**A**) Reference of combined Group SCC, (**B**) Reference of combined Group SWC, (**C**) Reference of combined Group NCC, (**D**) Reference of combined breeds XZC, Liangshan cattle (LSC), and Hannan cattle (HNC).

For scenario II, at a heritability of 0.6, the average accuracies of prediction for breeds in reference were larger than those that were not in reference. Figure 4 shows the accuracies of the prediction using three references, which comprised of breeds from each group. When combining three breeds from group SWC (Figure 4B), the average predictive accuracies using four strategies in groups (PWC, LSC, ZTC) were 0.53, 0.51, 0.53, and 0.51, while 0.13, 0.09, 0.09, and 0.09 were estimated for other breeds. When combined three breeds (XZC, LSC, HNC) as reference from different groups, we found the average predictive accuracies using four strategies in reference were 0.38, 0.34, 0.39, and 0.36, while the accuracies for other breeds were 0.09, 0.08, 0.06, and 0.09 (Figure 4D).

#### 3.2.3. Prediction with Combined-10-Breeds Reference

**Figure 5:**
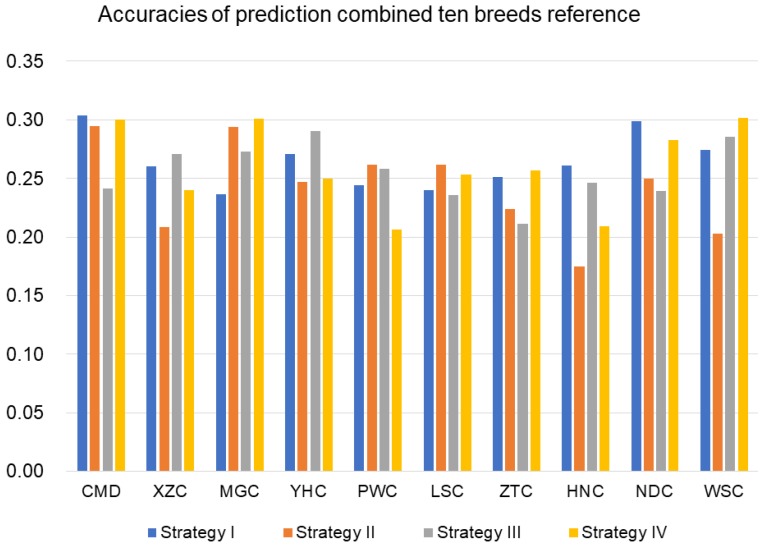
Accuracies of genomic prediction for 10 breeds using a combined reference population of 10 breeds for four traits with heritability (0.6).

Scenario III considered a reference population consisting of all 10 breeds. The average predictive accuracies in this scenario were 0.26, 0.24, 0.26, and 0.26 for four traits with a heritability of 0.6 (Figure 5). Predictive accuracies for *h*^2^ = 0.1 and *h*^2^ = 0.3 were presented in the Supplementary Material (Supplementary S2).

### 3.3. Effect of Heritability

**Figure 6:**
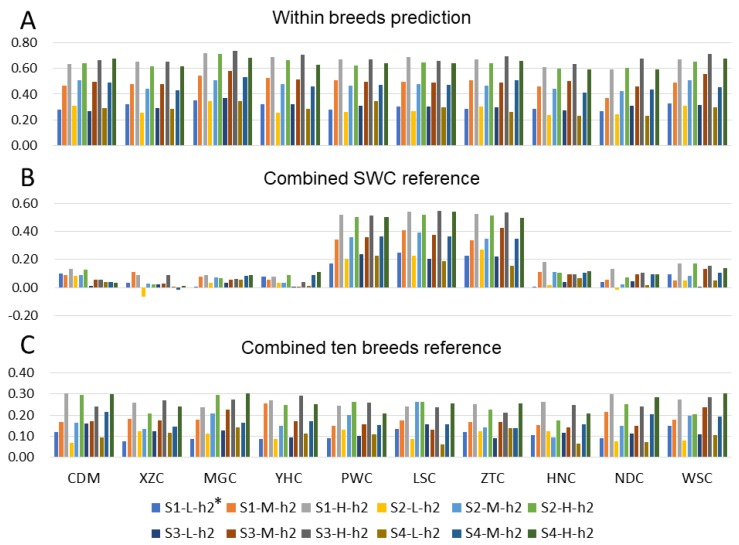
Accuracies of genomic prediction for 10 breeds using within-breed and a combined 10-breeds reference population for different heritability. *S1-L-h^2^: Low heritability (*h*^2^ = 0.1) in strategy I. *S2-H-h^2^: High heritability (*h*^2^ = 0.6) in strategy II.

We compared the consequences of alternative heritability of the traits. Figure 6 shows the effect of heritability on predictive accuracy for scenarios of within-breed, admixed reference, and 10-breeds mixed reference. When the heritability increased, the accuracy increased in all breeds for each scenario. The average accuracies of prediction for within-breed reference were 0.29, 0.48, and 0.66 for heritability 0.1, 0.3, and 0.6, respectively. When group SWC was the reference, the predictive accuracies for SWC were 0.21, 0.37, and 0.52, respectively, for three heritability values, while the average predictive accuracies for other breeds were 0.03, 0.07, and 0.10. For heritability 0.1, 0.3, and 0.6, the predictive average accuracies for 10 breeds were 0.11, 0.17, and 0.26 using the reference of combined 10 breeds.

### 3.4. Effect of Genetic Architecture

We also compared the results considering alternative genetic architecture of these traits. As shown in Table 2, the influence of the number of QTLs on the predictive accuracy for scenarios of within-breed and combined-10-breeds as reference. For within-breed, the average predictive accuracies for four traits with different heritability were 0.48, 0.46, 0.50, and 0.50, respectively. For 10 combined-breeds reference, the average predictive accuracies were 0.18, 0.17, 0.18, and 0.18.

## 4. Discussion

### 4.1. Simulation of Genotype and Phenotype

Evaluation of multiple-population prediction depends on the LD pattern of these populations. Thus, understanding LD pattern from different population can offer valuable insights into investigating the genomic prediction of multiple populations. In present study, to reflect the allele frequencies and population LD pattern manifested in real population, we performed simulations using a resampling approach proposed by [23,24]; the simulated genotype for each individual was produced by resampling genotype fragments from the real genotype of studied animals. Therefore, the simulated population retains the basic LD patterns and allele frequencies observed in the real data from Chinse indigenous cattle. Our results provide an important evident for the theoretical evaluation of genomic prediction for Chinse indigenous cattle.

Also, we evaluated the properties of genomic changes at genome-wide level and compared the performances of different strategies [33]. According to PCA results and persistence of phase analyses, we found that the simulated genotype can reflect the realistic LD pattern and is feasible for investigating the genomic prediction for multiple breeds.

For simulation, QTLs were randomly selected from SNPs loci in the real genotype data set. QTLs have different allele frequencies in different breeds; thus, most QTLs can be considered as being segregated in these breeds. The simulated trait for each individual among population was different due to the difference of MAF, which can reflect real data for different populations, and this strategy can facilitate the evaluation of genomic prediction for multiple population.

### 4.2. Predictive Accuracies from Admixed Population

In this study, we found the predictive accuracies were relatively low when the reference and validation populations sampled from different breeds. The reason may be because high LD (causes correlation between SNP and causal polymorphisms) existed within the breeds that were studied, while not in other breeds [34]. These results are consistent with previous empirical studies involving traits of similar heritability [1,35,36]. Meuwissen et al. found predictive reliability of 0.62 in a simulation analysis for a training set with 1000 phenotypes and a heritability of 0.5 [1]. The composition of the reference population had a large effect on the prediction accuracy, especially the relationships between reference and validation populations [37]. Our study suggested that the predictive accuracies were higher when these breeds were included in the reference population. As reported in a previous study, the accuracy of genomic prediction ranged from 0.01 to 0.19 in Holstein-Friesian and Jersey cows, and the accuracy was not significantly increased by adding individuals from other breeds to reference population [38]. In practice, pooling data from different breeds can increase the power of genetic gain when the components of admixture are genetically related. The predictive accuracies were higher when the combined multiple breeds were clustered in the same group according to K-means approach [26]. In this study, using reference SWC which were comprised of PWC, LSC, ZTC, the predictive accuracies for SWC were 0.47, 0.33, and 0.19 for high, middle, and low heritability, respectively. We found the accuracies were about 28.65%, 34.04%, and 27.11%, which were higher than the population combined of XZC, LSC, and HNC. Our results provided valuable insights into the application of multiple population selection regarding the pooled data approach. Moreover, several studies reported that the pooling data approach may decrease the predicative accuracies for the admixed population [39,40].

Our results agreed with previous findings, which suggested the predictive accuracies using seven combined breeds as reference were between 0.363 and 0.330 for heritability 0.4 in Spanish native cattle, and the accuracies decrease when heritability decreased [41]. The pooled data approach is likely to cause a decrease in accuracies, especially for the components of the admixed populations with small population size [10]. The main issue is how to bring additional benefits, considering the cost of genotyping. To increase the predictive accuracy of genomic breeding values, a large number of animals with both genotypes and phenotypes were required in training population [1,42]. Adding individuals from genetically related populations is useful when genotyping small populations. This strategy should be feasible for the application of genomic prediction for many small breeds, such as indigenous cattle in many countries.

### 4.3. Effect of Heritability and Genetic Architecture

The heritability and genetic architecture of the trait can influence the genetic gain for genomic prediction in breeding program [43,44]. In our study, the GBLUP was used to predict genetic merit, which was based on assumption that each marker have same effect [45]. The heritability of the phenotypes can affect the reliability of GEBVs [1,3,46], the predictive accuracies were low for traits with low heritability. In our simulation, the lower heritability causes lower predictive accuracies for most of traits and scenarios. The additive model with all common variants could recover only a fraction of the total heritability for complex traits [47], and the predictive accuracy being lower in real data can be explained by the missing heritability phenomenon caused by non-additive effects.

Previous studies suggested the genomic predictions from real data were not consistent with the results of simulation analysis [48,49]. One reason could be explained that the simulated data with various genetic architecture is significantly different from real populations. Many studies have compared methods using simulated genetic architectures with 50 or fewer QTLs, their finding revealed the genetic architecture can affect the accuracy of prediction, including number and variance QTLs [48,50].

In this study, we investigated different genetic architectures by simulating different numbers of QTLs. As shown in Figure 6, the lowest predictive accuracy using both multi-breed and single breed references was observed in phenotype simulation strategy II, few QTLs were simulated affecting the studied trait, and 25 QTLs can explain ~25% of genetic variance. The difference of MAF among breeds may result in negative prediction accuracies. These results may be caused by the inconsistent of QTL effects between breeds, and the weak LD level among them [50]. Similar results has also been found in a multi-breed genomic prediction, the across breed predictive accuracies were lower than within-breed prediction, and marker selection strategies can lead to more accurate genomic prediction in multiple small breeds and improve rate of genetic gain [51]. In general, the GBLUP models assume that variance and covariance of SNP are the same across the genome [52], while Bayes assumes that the distribution of SNP effects is a mixture of normal distributions [42]. Knowledge of the genetic architecture can improve the performance of genomic prediction using Bayesian models by assigning locus-specific priors to markers, therefore, Bayes models with locus-specific priors may increase the accuracy of across breed genomic predictions and should be considered in further studies.

## 5. Conclusions

Our study suggested that the application of meta-population can increase accuracy in scenarios with a reduced size of reference populations. Our findings also implied the potential application of a multiple-breed genomic selection in Chinese indigenous cattle.

## Figures and Tables

**Table 1:** Overview of phenotype simulation strategies.

Simulation Strategy	nQTL ^1^	nS ^2^	nM ^3^	nL ^4^	Heritability
I	100	0	0	100	0.1/0.3/0.6
II	2000	1361	614	25	0.1/0.3/0.6
III	5000	4595	390	15	0.1/0.3/0.6
IV	10,000	10,000	0	0	0.1/0.3/0.6

^1^ Total number of QTL. ^2^ Number of QTL with small effect (nS). ^3^ Number of QTL with medium effect (nM). ^4^ Number of QTL with large effect (nL).

**Table 2:** Comparison the effect of QTLs for accuracies for within-breed and combined-10-breeds estimation.

	Within Breed				Combined Breeds			
Strategy ^*^	I	II	III	IV	I	II	III	IV
L-h^2 *^	0.30	0.28	0.31	0.29	0.11	0.10	0.12	0.10
M-h^2 *^	0.48	0.47	0.51	0.47	0.18	0.17	0.17	0.17
H-h^2 *^	0.66	0.64	0.68	0.64	0.26	0.24	0.26	0.26
Average	0.48	0.46	0.50	0.47	0.18	0.17	0.18	0.18

L-h^2 *^: Low heritability (*h*^2^ = 0.1). M-h^2 *^: Medium heritability (*h*^2^ = 0.3). H-h^2 *^: High heritability (*h*^2^ = 0.6). Strategy*: Simulation strategy for traits with different genetic architecture I ~ IV.

## Supplementary Materials

The following are available online at https://www.mdpi.com/2076-2615/9/10/789/s1. The genotype data reported in this article are available upon request for research. Supplementary S1 Basic information for samples. Supplementary S2 Summary of predictive accuracies for each scenario.

## Abbreviations

The following abbreviations are used in this manuscript:

GEBV	Genomic estimated breeding values
QTL	Quantitative trait loci
SNP	Single nucleotide polymorphism
GBLUP	Genomic best linear unbiased prediction
GRM	Genomic relationship matrix
TBV	True breeding values
MCMC	Markov Chain Monte Carlo
PCA	Principal Component Analysis
MAF	Minor allele frequency
GS	Genomic selection
GWAS	Genome-wide association study
LD	Linkage disequilibrium
Ne	Effective population size
QC	Quality control
SD	Standard deviation
pi-hat	Proportion identity by descent
MGC	Mongolia cattle
YHC	Yanhuang cattle
CDM	Caidamu cattle
XZC	Xizang cattle
PWC	Pingwu cattle
LSC	Liangshan cattle
ZTC	Zhaotong cattle
WSC	Wenshan cattle
HNC	Hannan cattle
NDC	Nandan cattle
NCC	Including north Chinese cattle group (contains CDM, YHC, MGC and XZC)
SWC	Southwest Chinese cattle group (contains LSC, PWC and ZTC)
SCHC	South Chinese cattle group (contains HNC, NDC and WSC)
