# Supplementary material for: Theoretical Evaluation of Multi-Breed Genomic Prediction in Chinese Indigenous Cattle
Source: Animals (Basel). 2019 Oct 11;9(10):789. doi: 10.3390/ani9100789 (PMC6827096; doi:10.3390/ani9100789)
Supplement: Supplementary file 1 [file animals-09-00789-s001.zip › animals-572958-SI.pdf]

Table 1. Accuracies of within breed prediction.

Trait	Heritability	Validation									
		CDMC	XZC	MGC	YHC	PWC	LSC	ZTC	HNC	NDC	WSC
Strategy 1	0.1	0.28	0.32	0.35	0.32	0.28	0.30	0.28	0.29	0.27	0.32
	0.3	0.47	0.48	0.54	0.52	0.51	0.50	0.51	0.46	0.37	0.49
	0.6	0.63	0.66	0.72	0.69	0.67	0.69	0.67	0.61	0.59	0.67
Strategy 2	0.1	0.29	0.28	0.35	0.29	0.35	0.30	0.26	0.23	0.23	0.30
	0.3	0.49	0.43	0.53	0.46	0.47	0.47	0.51	0.41	0.44	0.45
	0.6	0.68	0.62	0.68	0.63	0.64	0.64	0.66	0.59	0.59	0.68
Strategy 3	0.1	0.27	0.29	0.37	0.32	0.31	0.31	0.30	0.27	0.31	0.32
	0.3	0.50	0.48	0.58	0.51	0.50	0.49	0.49	0.50	0.46	0.56
	0.6	0.67	0.65	0.74	0.71	0.67	0.66	0.69	0.64	0.67	0.71
Strategy 4	0.1	0.31	0.25	0.34	0.25	0.26	0.27	0.30	0.24	0.24	0.31
	0.3	0.51	0.44	0.51	0.48	0.47	0.48	0.47	0.44	0.43	0.51
	0.6	0.64	0.61	0.71	0.67	0.62	0.65	0.64	0.60	0.60	0.65

Table 2. Accuracies of predict of single breed reference (PWC).

Trait	Heritability	Validation									
		CDMC	XZC	MGC	YHC	PWC	LSC	ZTC	HNC	NDC	WSC
Strategy 1	0.1	0.05	0.05	-0.04	0.03	**0.28**	0.06	-0.01	0.03	0.10	0.02
	0.3	0.07	0.07	0.08	0.07	**0.51**	0.10	0.06	0.13	0.12	-0.01
	0.6	0.11	0.06	0.04	0.07	**0.67**	0.11	0.05	0.12	0.16	0.06
Strategy 2	0.1	0.07	0.02	0.04	-0.01	**0.35**	0.04	0.01	0.04	0.02	0.07
	0.3	0.09	0.06	0.05	0.01	**0.47**	0.02	0.07	0.02	0.08	0.08
	0.6	0.05	0.00	0.05	0.02	**0.64**	0.09	0.06	0.04	0.07	0.13
Strategy 3	0.1	-0.02	0.04	0.03	0.02	**0.31**	0.06	-0.03	0.09	0.03	0.00
	0.3	0.01	0.02	0.12	0.01	**0.50**	0.05	0.03	0.06	0.10	0.09
	0.6	0.06	0.04	0.09	0.04	**0.67**	0.09	0.06	0.08	0.10	0.09
Strategy 4	0.1	0.00	-0.03	0.03	0.03	**0.26**	0.07	0.01	0.07	0.05	0.06
	0.3	0.04	0.06	0.04	0.02	**0.47**	0.09	0.08	0.07	-0.06	0.04
	0.6	0.04	0.02	0.03	0.04	**0.62**	0.12	0.05	0.07	0.03	0.07

Table 3. Accuracies of predict with combind reference of NCC group (CDMC, XZC, MGC and YHC).

Trait	Heritability	Validation									
		CDMC	XZC	MGC	YHC	PWC	LSC	ZTC	HNC	NDC	WSC
Strategy 1	0.1	0.17	0.12	0.14	0.14	0.02	0.04	0.03	0.02	0.02	0.00
	0.3	0.27	0.27	0.28	0.28	0.00	0.08	0.09	0.04	0.01	0.05
	0.6	0.40	0.37	0.42	0.39	0.05	0.06	0.13	0.09	0.04	0.10
Strategy 2	0.1	0.15	0.19	0.21	0.13	0.02	0.05	0.10	0.07	0.03	0.09
	0.3	0.26	0.28	0.26	0.23	0.05	0.06	0.05	0.08	0.07	0.03
	0.6	0.40	0.35	0.41	0.39	0.12	0.10	0.08	0.09	0.08	0.05
Strategy 3	0.1	0.16	0.08	0.21	0.19	0.08	0.05	0.02	0.10	0.00	0.01

	0.3	0.23	0.28	0.32	0.31	0.15	0.06	0.04	0.10	0.02	0.05
	0.6	0.41	0.39	0.44	0.40	0.16	0.04	0.05	0.14	0.04	0.07
	0.1	0.16	0.11	0.20	0.13	-0.02	0.03	0.04	0.03	0.05	0.00
Strategy 4	0.3	0.24	0.23	0.31	0.29	0.06	0.05	0.05	0.02	0.08	0.08
	0.6	0.38	0.31	0.41	0.40	0.05	0.07	0.13	0.06	0.08	0.08

Table 4. Accuracies of predict with combind reference of SWC group (PWC, LSC and ZTC).

Trait	Heritability	Validation									
		CDMC	XZC	MGC	YHC	PWC	LSC	ZTC	HNC	NDC	WSC
	0.1	0.10	0.03	0.01	0.08	0.17	0.25	0.22	0.01	0.04	0.09
Strategy 1	0.3	0.09	0.11	0.08	0.05	0.34	0.41	0.33	0.11	0.06	0.05
	0.6	0.14	0.09	0.09	0.08	0.52	0.54	0.52	0.18	0.13	0.17
	0.1	0.04	0.01	0.06	0.01	0.23	0.19	0.16	0.07	0.02	0.05
Strategy 2	0.3	0.04	-0.02	0.08	0.09	0.37	0.37	0.35	0.10	0.10	0.10
	0.6	0.03	0.01	0.09	0.11	0.50	0.54	0.49	0.11	0.10	0.14
	0.1	0.01	0.02	0.03	0.01	0.24	0.20	0.22	0.04	0.05	0.00
Strategy 3	0.3	0.06	0.03	0.06	0.01	0.36	0.38	0.43	0.09	0.10	0.13
	0.6	0.06	0.09	0.06	0.04	0.52	0.54	0.54	0.09	0.11	0.15
	0.1	0.08	-0.07	0.03	0.03	0.21	0.23	0.27	0.01	-0.02	0.05
Strategy 4	0.3	0.09	0.03	0.07	0.03	0.36	0.39	0.35	0.11	0.02	0.08
	0.6	0.13	0.02	0.07	0.09	0.50	0.52	0.51	0.11	0.07	0.17

Table 5. Accuracies of predict with combind reference of SCHC group (HNC, NDC and WSC).

Trait	Heritability	Validation									
		CDMC	XZC	MGC	YHC	PWC	LSC	ZTC	HNC	NDC	WSC
	0.1	0.10	0.02	0.03	0.03	0.01	0.05	0.04	0.24	0.16	0.27
Strategy 1	0.3	0.03	0.03	0.11	0.01	0.06	0.09	0.05	0.38	0.31	0.40
	0.6	0.07	0.07	0.11	0.03	0.13	0.13	0.08	0.50	0.51	0.55
	0.1	0.02	0.03	0.00	0.03	0.04	0.06	0.00	0.16	0.21	0.20
Strategy 2	0.3	0.04	-0.01	0.03	0.10	0.10	0.02	0.09	0.33	0.36	0.36
	0.6	0.08	0.02	0.09	0.10	0.12	0.11	0.11	0.46	0.50	0.48
	0.1	0.04	0.01	0.06	0.01	0.01	0.07	0.05	0.21	0.19	0.25
Strategy 3	0.3	-0.01	0.04	0.05	0.01	0.02	0.11	0.08	0.40	0.36	0.41
	0.6	0.07	0.04	0.03	0.04	0.07	0.16	0.09	0.52	0.48	0.58
	0.1	0.08	-0.04	0.02	0.04	-0.03	0.04	0.03	0.14	0.23	0.28
Strategy 4	0.3	0.07	0.05	0.03	0.01	0.06	0.14	0.09	0.32	0.31	0.40
	0.6	0.11	0.06	0.08	0.04	0.08	0.09	0.10	0.44	0.46	0.52

Table 6. Accuracies of predict with combind reference among group (XZC, LSC and HNC).

Trait	Heritability	Validation									
		CDMC	XZC	MGC	YHC	PWC	LSC	ZTC	HNC	NDC	WSC

Strategy 1	0.1	-0.01	0.12	0.03	0.02	0.07	0.15	0.03	0.16	0.03	0.03
	0.3	0.04	0.26	-0.01	0.06	0.05	0.26	0.06	0.29	0.10	0.02
	0.6	0.05	0.36	0.08	0.10	0.11	0.37	0.15	0.40	0.07	0.08
Strategy 2	0.1	0.03	0.18	-0.01	0.04	0.04	0.11	0.04	0.18	0.02	0.04
	0.3	0.02	0.28	0.03	0.06	0.03	0.25	0.02	0.26	0.09	0.02
	0.6	0.06	0.35	0.09	0.09	0.08	0.39	0.06	0.33	0.13	0.13
Strategy 3	0.1	0.08	0.09	0.01	0.04	0.09	0.19	0.02	0.15	0.00	0.06
	0.3	0.08	0.27	0.00	0.06	0.13	0.29	0.01	0.25	0.03	0.14
	0.6	0.07	0.38	0.04	0.08	0.10	0.39	0.01	0.39	0.02	0.11
Strategy 4	0.1	0.02	0.11	0.04	0.06	-0.02	0.16	0.04	0.13	0.03	-0.02
	0.3	0.03	0.23	0.05	0.05	0.05	0.31	0.04	0.19	0.03	0.06
	0.6	0.08	0.31	0.08	0.06	0.07	0.36	0.10	0.36	0.07	0.11

Table 7. Accuracies of predict with combind ten breeds reference.

Trait	Heritability	Validation									
		CDMC	XZC	MGC	YHC	PWC	LSC	ZTC	HNC	NDC	WSC
Strategy 1	0.1	0.12	0.08	0.09	0.09	0.09	0.13	0.12	0.11	0.09	0.15
	0.3	0.17	0.18	0.18	0.25	0.15	0.17	0.17	0.15	0.21	0.18
	0.6	0.30	0.26	0.24	0.27	0.24	0.24	0.25	0.26	0.30	0.27
Strategy 2	0.1	0.09	0.12	0.14	0.11	0.11	0.06	0.14	0.06	0.07	0.11
	0.3	0.22	0.15	0.16	0.17	0.15	0.16	0.14	0.16	0.20	0.19
	0.6	0.30	0.24	0.30	0.25	0.21	0.25	0.26	0.21	0.28	0.30
Strategy 3	0.1	0.16	0.12	0.13	0.09	0.10	0.15	0.09	0.12	0.11	0.11
	0.3	0.17	0.17	0.22	0.17	0.15	0.13	0.17	0.14	0.15	0.24
	0.6	0.24	0.27	0.27	0.29	0.26	0.24	0.21	0.25	0.24	0.29
Strategy 4	0.1	0.07	0.12	0.11	0.09	0.13	0.09	0.12	0.12	0.08	0.08
	0.3	0.16	0.14	0.21	0.15	0.20	0.26	0.14	0.09	0.15	0.20
	0.6	0.29	0.21	0.29	0.25	0.26	0.26	0.22	0.18	0.25	0.20
